# Kaposi of the penis: about a case

**DOI:** 10.11604/pamj.2019.32.137.18274

**Published:** 2019-03-22

**Authors:** Sqalli Houssaini Asmaa, Badreddine Hassam

**Affiliations:** 1 Dermatology and Venerology Department, University Hospital Center Ibn Sina, Faculty of Medicine and Pharmacy, Mohammed V University, Rabat, Morocco

**Keywords:** Kaposi, glans, retroviral infection

## Image in medicine

Kaposi sarcoma (KS) is a multicentric angioproliferative disorder of endothelial origin. It predominantly affects mucocutaneous sites but may also affect visceral organs. We are reporting a case of KS with unusual presentation. A 45-year-old male presented violaceous macules on the glans which appeared 3 weeks ago. Otherwise he reported fever and loss weight. Histological examination detected the presence of spindle cells and the positivity for Kaposi-associated Herpes virus-8 confirmed the lesions as Kaposi's sarcoma. Visceral sarcoma lesions were also present in the oesophagus and gastric fundus. HIV viral serology was positive. Treatment with antiretroviral therapy and bleomycine was initiated. Sarcomas of the penis are very uncommon, representing less than 5% of malignant tumors in this area. KS is the most common sarcoma of the penis and the second most common is leiomyosarcoma. Primary presentation of KS on the penis is not common but more often observed in patients with AIDs, whose lesions are the aggressive form, and only approximately 2-3% cases have shown penile KS lesions as first manifestation of disease as our patient. This case is important as it illustrates that disseminated KS was not to be predicted by the number or the external lesions.

**Figure 1:**
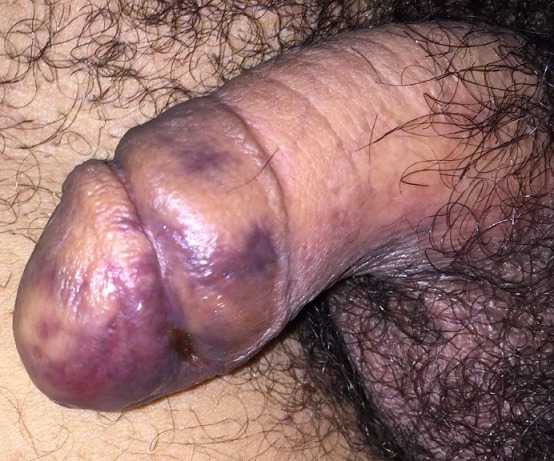
A violaceous macules on the glans

